# Effects of Shot Peening on Fretting Fatigue Crack Initiation Behavior

**DOI:** 10.3390/ma12050743

**Published:** 2019-03-04

**Authors:** Xin Liu, Jinxiang Liu, Zhengxing Zuo, Huayang Zhang

**Affiliations:** 1 School of Mechanical Engineering, Beijing Institute of Technology, Beijing 100081, China; LX_9002@163.com (X.L.); zxzuo@bit.edu.cn (Z.Z.); 2 School of Aeronautical Engineering, Zhengzhou University of Aeronautics, Zhengzhou 450046, China; zhhy2009bit@163.com

**Keywords:** fretting loading, crack initiation, shot peening, residual stress relaxation

## Abstract

This study analyzes the effects of shot peening on the crack initiation behavior under fretting loading by using a numerical method. The residual stress relaxation and the contact stress evolution are both considered. The crack initiation life is predicted by the critical plane Smith–Watson–Topper (SWT) model. Considering that the fretting contact region has a high stress gradient along the depth direction, the process volume approach is adopted to calculate the SWT parameters. The results show that the remaining residual stress after relaxation strongly affects crack initiation life. The remaining residual stress decreases with the increase of fatigue loading, and the effect of shot peening on the improvement of crack initiation life is more obvious under smaller fatigue loading. Furthermore, under smaller fatigue loading, the crack initiation life of specimens with high shot peening intensity is longer than that of specimens with low shot peening intensity. However, the opposite phenomenon appears when the fatigue loading is large enough.

## 1. Introduction

Fretting caused by cyclic contact stresses can occur at any place when there is a small relative motion between two components [1]. Under cyclic contact stresses, fretting facilitates the premature initiation of fatigue cracks due to stress concentration, which leads to a shorter fatigue life of the components [2,3]. For fretting fatigue, the cyclic contact stresses acting on the component are composed of contact pressure and contact shear stress. Therefore, the working stress produced by fretting has a great gradient along the depth direction, which is an important difference between fretting fatigue and plain fatigue. In the field of engineering machinery, it is possible to effectively improve the fatigue life of components by shot peening [4,5,6]. The residual stress introduced by shot peening can suppress the initiation of fatigue cracks, and the fatigue life of components is enhanced [7,8]. The distribution of residual stress usually increases first and then decreases along the depth direction. Thus, the superposition of residual stress and working stress produces a complex stress state and high stress gradient. Furthermore, the residual stress is not constant during the fretting process, and it can be released under cyclic contact stresses and fatigue bulk stress. In addition to introducing residual stress, shot peening also changes the wear resistance of materials [9]. With different wear resistances, the variation of the wear profile with an increasing number of loading cycles is different under the same loading conditions. The wear profile directly determines the distribution of contact stresses. Thus, the evolution of contact stresses is different for the components with different shot peening. Under fretting conditions, such a complex stress environment makes the study on the effects of shot peening on crack initiation behavior more difficult.

At present, the effects of shot peening on fretting fatigue crack initiation behavior have been investigated by many scholars. By means of experimental methods, Yang et al. found that shot peening greatly reduced the type and number of crack initiation [10]. According to experimental and numerical results, Majzoobi et al. found that the effect of shot peening on the improvement of crack initiation life depended on the bulk stress level [4]. Through an experimental study, Li et al. found that the wet peening samples exhibited better resistance to fretting fatigue due to the synergistic effect of enhanced hardness and induced compressive residual stress compared to unpeened samples [11]. The experimental results of Liu et al. showed that at higher fatigue stress levels, the fretting fatigue crack initiation sites were on the surface for the shot-peened specimens. However, at lower fatigue stress levels, the crack initiation sites were on the subsurface [12]. Although crack initiations can be observed via experimental methods, considering that the cracks are usually covered by fretting pads, it is difficult to estimate the precise crack initiation moment. In addition to experimental methods, numerical methods are also used to investigate the fretting fatigue crack initiation behavior for shot-peened components. Sabelkin et al. used finite element method to analyze the fretting fatigue crack initiation life for the Ti-6Al-4V material with different shot peening intensities [13]. It was found that the improvement of the fatigue crack initiation life was directly related to the magnitude of residual stress. Vázquez et al. proposed a multiaxial fatigue life model that considered the presence and cylic relaxation of a residual stress filed [14]. Gangaraj et al. pointed out the inconsistency between residual stress and working stress distribution under fretting conditions [15]. However, the crack initiation life of the shot-peened specimen was not predicted. Although some papers regarding fretting fatigue crack initiation behavior of shot-peened specimen exist, the effects of residual stress relaxation on crack initiation behavior remain elusive. Therefore, the effects of shot peening on fretting fatigue crack initiation behavior considering residual stress relaxation needs to be further researched.

In this paper, a two-dimension cylinder-plate contact finite element model is used to study the effects of shot peening on the fretting fatigue crack initiation behavior. The simulation of residual stress relaxation considers the change of surface yield strength. The evolution of contact surface profile caused by fretting wear is described by the Archard equation [16]. The complicated stress state and the high stress gradient are considered by using the process volume approach. Finally, the effects of shot peening on the fretting fatigue crack initiation life and crack initiation location are found.

## 2. Effects of Shot Peening on Material

Shot peening is an advanced surface treatment process to introduce the residual stress into the material surface. In addition to residual stress, shot peening also changes the yield strength and the wear resistance of the material surface. These changed material properties are important parameters influencing the mechanical behavior and the stress state of materials. In order to comprehensively consider the effects of shot peening on the material surface in the study of fretting fatigue crack initiation behavior, the residual stress, yield strength, and wear resistance are obtained from the same shot-peened specimen through experimental methods.

In this paper, the fatigue specimens are made of aluminum 7075-T6 alloy plate with the following chemical compositions (weight per cent %): 6.1 Al, 0.01 Zn, 0.4 Ti, 0.16 Si, 2.8 Mn, 0.4 Fe, 2 Cu, 0.18 Cr, and Al for the remainder. The stress-strain relationship of aluminum 7075-T6 is measured by the monotonic tensile experiment, and the obtained material properties are shown in Table 1.

**Figure 1:**
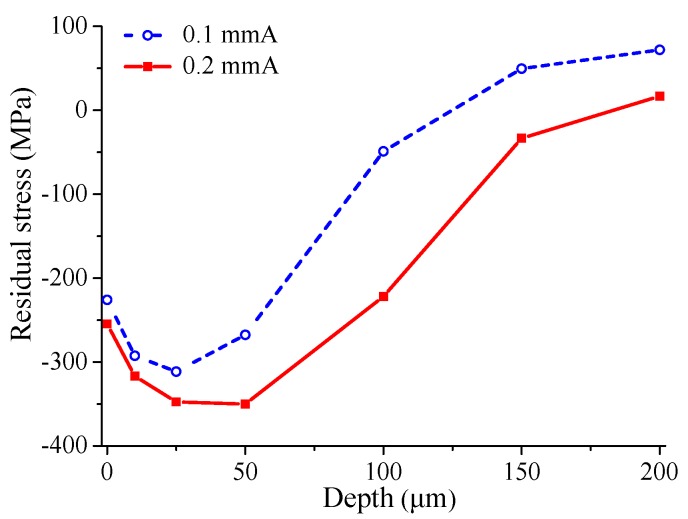
Residual stress versus depth for different shot-peened specimens.

Two different shot peening intensities, namely 0.1 mmA and 0.2 mmA, are adopted in this study. The residual stresses introduced by shot peening are measured by the X-ray diffraction experiments, and the experimental instrument is X-350A X-ray diffractometer (Handan Stress Technologies Co. Ltd., China). The experiments are conducted with the $\sin_{\psi }^{2}$ method by using CrKα radiation from the (311) diffraction plane of the FCC (Face Center Cubic) structure. Furthermore, the subsurface residual stress measurements are conducted after each successive layer removal by electrolytic polishing. Electrolytic layer removal is carried out in steps to a depth of approximately 200 μm. The measurement results of residual stress along the material depth direction are shown in Figure 1. It can be seen that the residual compressive stresses along the depth direction first increase to a maximum value, and then gradually decrease. Furthermore, the higher shot peening intensity has the greater value of residual compressive stress at the same depth.

**Figure 2:**
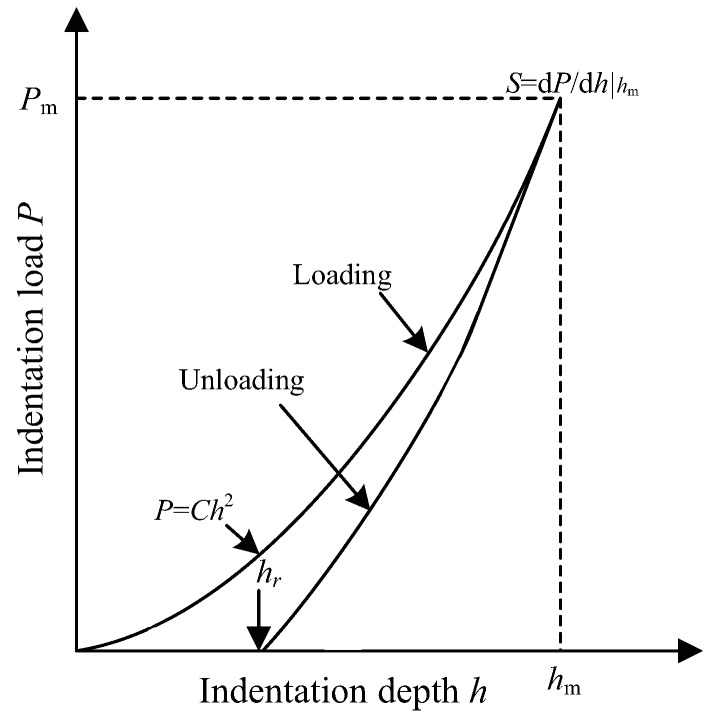
Schematic illustration of a typical indentation load-depth (*P*-*h*) curve.

**Figure 3:**
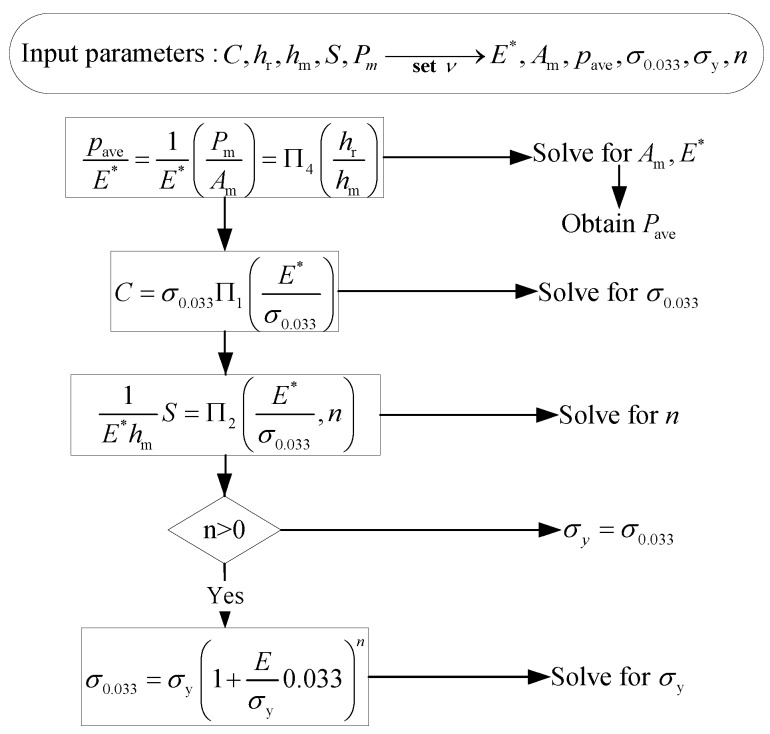
The flow chart of Dao’s analysis algorithms [18].

**Figure 4:**
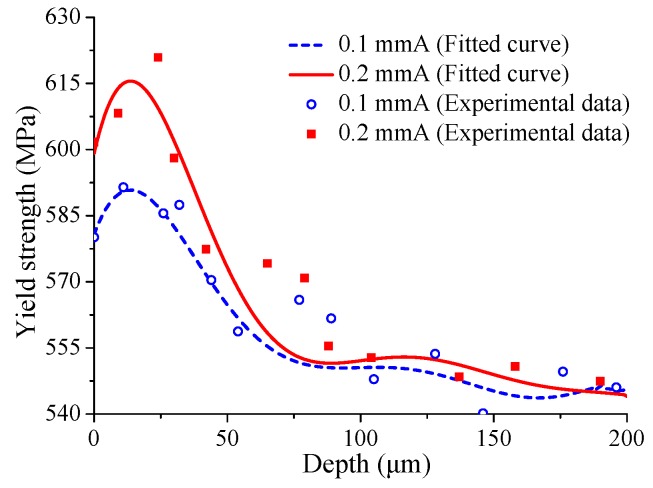
Yield strength versus depth for different shot-peened specimens.

In addition to introducing residual stress, shot peening also introduces initial plastic deformation. The initial plastic deformation can improve the in-plane tensile yield strength of the material surface layer [17]. In this study, the tensile yield strength of shot-peened material surface layer is obtained by the nano-indentation experiments. The experimental maximum indentation depth is 2000 nm, and the surface approach velocity is 10 nm/s. Figure 2 shows a typical indentation load-depth curve. By using the relationship between indentation load and indentation depth measured by the experiments, the characteristic parameters of loading curvature *C*, unloading contact stiffness *S*, maximum indentation depth $h_{m}$, maximum indentation load $P_{m}$, and the residual depth of penetration after complete unloading $h_{r}$ can be obtained. Then, the yield strength $\sigma_{y}$ is calculated by the aforementioned characteristic parameters combined with the dimensional analysis method of Dao [18]. The flow chart of Dao’s analysis algorithm is shown in Figure 3. Where $\sigma_{0.033}$ is the stress corresponding to plastic strain $\varepsilon_{p}=0.033$, $A_{m}$ stands for the indentation contact area, and $p_{\mathrm{ave}}$ = $p_{m}$/$A_{\mathrm{ave}}$. $\prod_{1}$, $\prod_{2}$, and $\prod_{4}$ are universal dimensionless functions, and the analytic formulations of the functions are presented in reference [18]. Furthermore, in order to measure subsurface yield strength along the depth direction, the surface layer removal is carried out in steps of about 10–20 μm to a depth of 200 μm. The measurement results of yield strength along the depth direction are shown in Figure 4. It can be seen from the experimental results that the yield strength first increases to a maximum value, and then gradually decreases until it stabilizes at about 545 MPa below 80 μm depth. The yield strength of the unpeened specimen measured by monotonic tensile experiment is 545 MPa. It indicates that the subsurface material of shot-peened specimens below 80 μm has almost no plastic deformation. Moreover, the main differences between the two yield strength profiles are the surface yield strength and the maximum subsurface yield strength. Higher shot peening intensity leads to a greater value for surface yield strength and maximum subsurface yield strength. In addition, the fitting curves of the experimental results are also shown in Figure 4, and can be expressed as:

(1) $$\sigma_{y}=a_{1}\sin\left(b_{1}h+c_{1}\right)+a_{2}\sin\left(b_{2}h+c_{2}\right)+a_{3}\sin\left(b_{3}h+c_{3}\right)+a_{4}\sin\left(b_{4}h+c_{4}\right).$$

The variables in Equation (1) are yield strength $\sigma_{y}$ and material depth *h*. The coefficients in Equation (1) are $a_{n}$, $b_{n}$, and $c_{n}$, and the values of coefficients are listed in Table 2. As can be seen from the figure, the fitting curves are in good agreement with experimental results. More precisely, the maximum error between experimental results and fitting curves is only about 2.8%.

**Figure 5:**
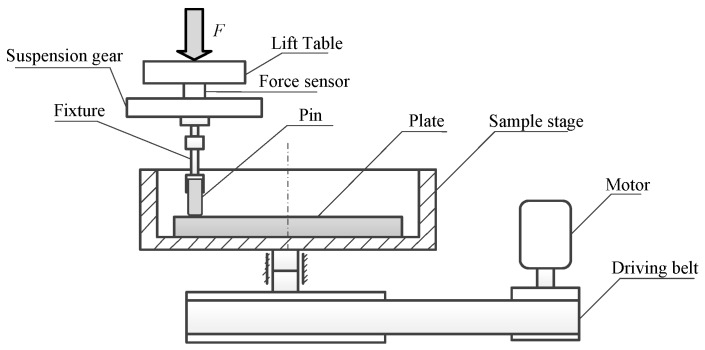
Pin-on-plate rotating wear experiment device.

**Figure 6:**
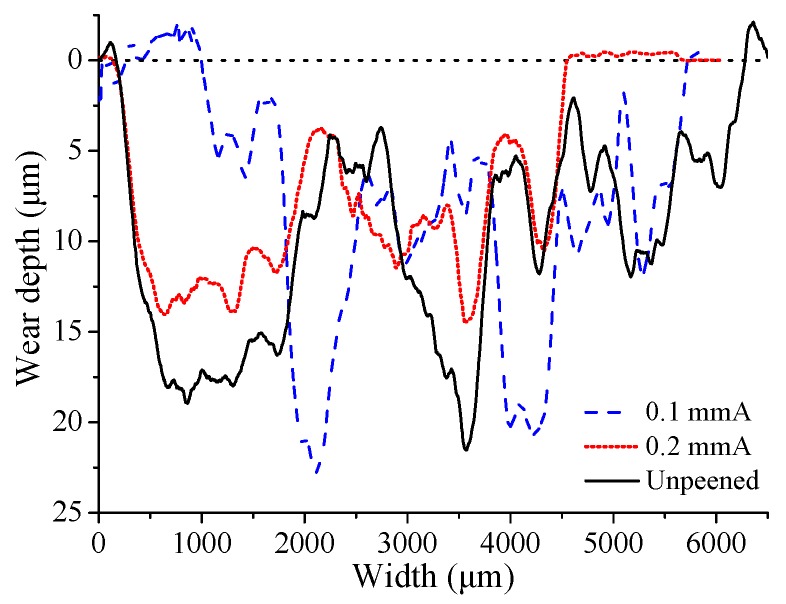
The section of wear scar measured by 3D surface profiler.

The wear resistance of material can be evaluated by the Archard wear coefficient *k* [16], which is defined by:

(2) $$k=\frac{V}{\delta \cdot F}.$$

where *V* represents the total wear volume, δ stands for the total accumulated displacement, *F* is the applied normal force. In this study, the Archard wear coefficient of the aluminum 7075-T6 is measured by the pin-on-plate rotating wear experiments [19]. The wear experiment adopts the UMT-2 friction and wear experimental device, and the schematic of experimental device is shown in Figure 5. The plate specimen is aluminum 7075-T6, and the pin specimen is GCr15 steel with stronger wear resistance. The influence of surface roughness is eliminated by mirror polishing. The experimental normal force *F* is 50 N, the rotating speed *v* is 200 rpm, and the radius of rotation motion *r* is 20 mm. The experiment runs for 80 min at room temperature. After wear experiments, the wear scars are measured by 3D surface profiler, and the measured sections of wear scars are shown in Figure 6. The total wear volume *V* can be approximately estimated as the section area of wear scar $S_{a}$ times the perimeter of rotation motion $C_{P}$. The total accumulated displacement δ is equal to the rotating speed *v* multiplied by the running time *t* and multiplied by the perimeter $C_{P}$. Thus, the results show that the Archard wear coefficients of the 0.1 mmA and 0.2 mmA shot-peened aluminum 7075-T6 specimens are $5.83\times 10^{-8}$ ${\mathrm{MPa}}^{-1}$ and $5.04\times 10^{-8}$ ${\mathrm{MPa}}^{-1}$, respectively. Furthermore, the Archard wear coefficient of the unpeened aluminum 7075-T6 specimen is $8.011\times 10^{-8}$ ${\mathrm{MPa}}^{-1}$. By comparison, it can be found that the wear resistance of the aluminum 7075-T6 specimen is improved after shot peening, and the shot peening with higher intensity further improves wear resistance.

## 3. Simulation Model

**Figure 7:**
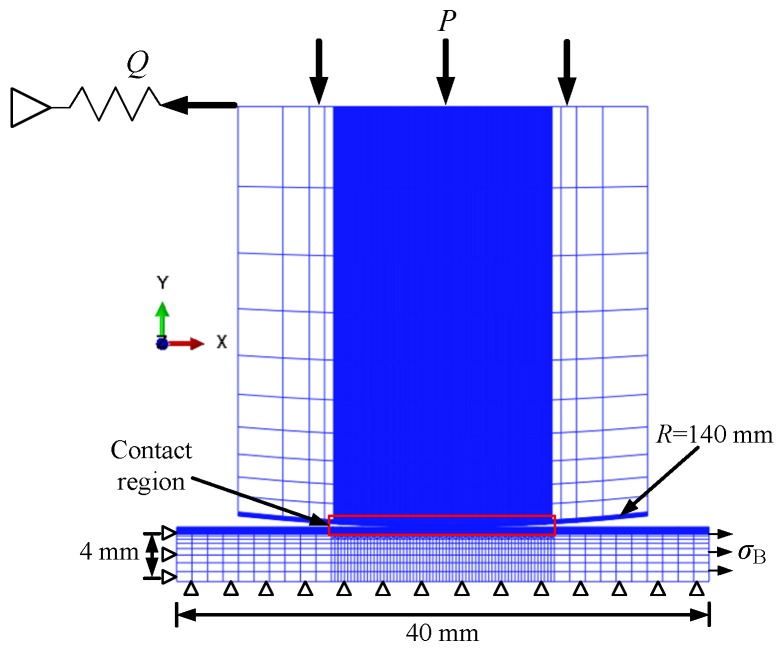
Two-dimensional fretting fatigue finite element model.

In this paper, a typical cylinder-plate contact configuration is adopted to study the effects of shot peening on the crack initiation behavior under fretting conditions. As shown in Figure 7, a 2-D fretting fatigue finite element model is built with the commercial finite element software ABAQUS. In the figure, a constant normal pressure *P* is applied on the top of the cylinder pad to establish contact between pad and specimen, and a sinusoidal fatigue loading $\sigma_{B}$ is applied on the right end of the specimen to produce relative slip. For the pad, the *x* direction displacement is hindered by the spring force. For the specimen, the left end is fixed, and a symmetry constraint is applied to the bottom of the specimen. The specimen and pad adopt 21,880 quadrilateral 4-node plain strain elements (CPE4). In order to capture high stress gradient and ensure computational efficiency, the element size in the contact region is refined to 0.03 mm × 0.03 mm. In addition, a two-node spring element (SPRING2) is adopted to produce the tangential force *Q*.

In this study, the three fatigue loading levels 350 MPa, 400 MPa, 450 MPa are considered to analyze the effects of shot peening on the crack initiation behavior, and the stress ratio *R* is −1. The normal pressure applied on the top of the pad is 4 MPa. The friction coefficient between the GCr15 steel pad and the aluminum 7075-T6 specimen is set to 0.6, which is measured by the fretting fatigue experiments. By using the monotonic tensile experiment, it can be obtained that the Young’s modulus *E* of aluminum 7075-T6 specimen is 72,000 MPa, the Poisson’s ratio ν is 0.345, and the initial yield strength $\sigma_{y}$ is 545 MPa. The Young’s modulus *E* of GCr15 steel fretting pad is 219,000 MPa and the Poisson’s ratio ν is 0.3 [20].

The cyclic characteristic of aluminum 7075-T6 can be described as a simple kinematic hardening rule [21]. Under kinematic hardening condition, the change in yield strength can be described by the variation of back stress. The variation of back stress is obtained by the difference between yield strength of shot-peened surface layer and yield strength of as-received material. By using the ABAQUS user subroutine HARDINI, the back stress versus depth data is incorporated into the finite element model. Thus, the change in yield strength is assigned to the FE model.

With the help of ABAQUS user subroutine SIGINI, the residual stress profile measured by the X-ray experiments is incorporated into the FE model. The residual stress introduced into the FE model will occur the phenomenon of self-balancing. By comparing the residual stress after self-balanced and initial residual stress, it can be proved that the residual stress profile measured by the X-ray experiments has high accuracy.

The Archard wear model is commonly used to simulate fretting wear behavior [22], and its expression is as follows:

(3) dh=kpdδ.

where *k* is the wear coefficient, *p* represents the local contact pressure, dδ stands for the relative slip increment. Combined with the Archard wear model and adaptive mesh technology, the simulation of fretting wear behavior is implemented by using the UMESHMOTION user subroutine in ABAQUS.

Fretting wear behavior is usually uneven on the contact surface for cylinder-plate contact configuration. In order to simulate the uneven change, the mesh of the contact area usually needs to be divided very finely, and the simulation will consume a lot of computational memory and time. Considering that the fretting wear behavior needs to be simulated many cycles, it is necessary to improve the computational efficiency. Therefore, a cycle jumping technique [23] is adopted in this study. The cycle jumping technique assumes that the wear depth increment is constant over a given cycle jump size ΔN. Thus, the cumulative wear depth increment of ΔN cycles can be simulated by one calculation step, and the nodal wear depth increment Δh is calculated by the Equation (4).

(4) Δh=ΔNkp(x)dδ.

In order to improve the calculation efficiency under the premise of ensuring the calculation accuracy, a compromise is reached by using the cycle jump size ΔN of 250.

Considering that the fretting is a multiaxial fatigue phenomenon, the critical plane Smith–Watson–Topper (SWT) model [24] is adopted to predict the fretting fatigue crack initiation behavior. The model is based on the experimental observations that the fatigue crack occurs at a specified plane, and the fatigue life is controlled by the stress and strain amplitude on the specified plane. Thus, the SWT model can be expressed as follows:

(5) $$\;\mathrm{SWT}=\sigma_{\max}\frac{\Delta \varepsilon }{2}=\frac{{\sigma_{f}^{\prime }}^{2}}{E}{\left(2N_{f}\right)}^{2b}+\sigma_{f}^{\prime }\varepsilon_{f}^{\prime }{\left(2N_{f}\right)}^{b+c}\;$$

where $\sigma_{\max}$ stands for the maximum normal stress in the plane of principal strain range, Δε is the maximum principal strain range, $\sigma_{f}^{\prime }$ denotes the fatigue strength coefficient, $\varepsilon_{f}^{\prime }$ represents the fatigue ductility coefficient, *b* is the fatigue strength exponent, and *c* is the fatigue ductility exponent. The material parameters of the SWT model for aluminum 7075-T6 is shown in Table 3 [25].

Due to material removal, the stress and strain in the contact area are constantly changing with the increase of cycle number. Therefore, the damage caused by the fretting loading on the specimen is different in each cycle. Thus, a cumulative damage approach is required to consider the evolution of the stress and strain. In this paper, the most commonly used damage accumulation model Miner–Palmgren (M–P) rule is adopted [26]. Combined with the cycle jumping technique described above, the M–P rule can be expressed as Equation (6):

(6) $$\;W=\sum_{i=1}^{N_{t}/\Delta N}\frac{\Delta N}{N_{fi}}\;$$

where, *W* represents the accumulated damage, ΔN stands for the cycle jump factor, $N_{fi}$ is the predicted fretting cycles to crack initiation at loading cycles *i* by using critical plane SWT model, $N_{t}$ denotes the total number of fretting fatigue cycles. In the finite element simulation, the $N_{fi}$ is calculated for all plane orientations at intervals of one degree, and the material damage *W* accumulates on each plane. Thus, when the accumulated damage *W* of a plane reaches 1, crack initiation is assumed to have occurred.

**Figure 8:**
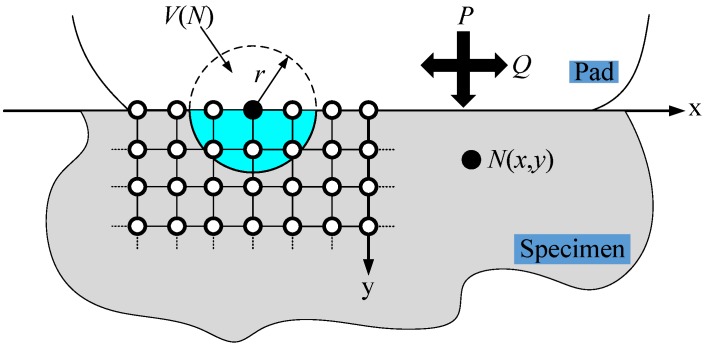
Schematic of radial shape process volume approach.

The life prediction results based on the stress of single danger point will seriously underestimate fretting fatigue crack initiation life [27], therefore, the effect of stress gradient must be considered. In this paper, a process volume approach [28,29] is adopted to consider stress gradient. In this approach, the stresses and strains are averaged over a specified radial shape volume, and then the SWT parameters are computed from these averaged values. As shown in Figure 8, the center of the radial shape volume is located at a node N(x,y) on the contact surface of the specimen. Search all the nodes in the range with N(x,y) as the origin and *r* as the radius on the specimen. Then, the crack initiation parameters of node N(x,y) are calculated by the average stresses and average strains of these nodes. Thus, the fretting fatigue crack initiation behavior depends on the degree of gradient change of the stress and strain within a certain critical volume range rather than just the stress and strain value at a certain point. The volume value is usually related to the grain size of the material. According to the experimental results, it can be known that the radius of radial shape volume for aluminum 7075-T6 is 80 μm [29].

By using the user subroutine UMAT in ABAQUS, the critical plane SWT model, the damage accumulation model, and the process volume approach are implemented in the finite element analysis model. In order to further improve the computational efficiency, only the refinement elements in the contact area are used to calculate the accumulated damage. When the accumulated damage of the integration point on a certain element reaches 1, the calculation stops. In this way, we can get the crack initiation life and crack initiation location.

## 4. Results and Discussion

**Figure 9:**
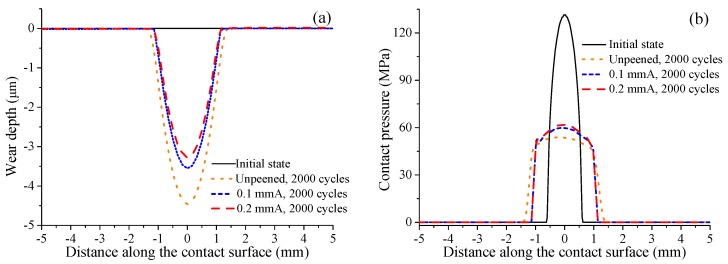
Distribution of (**a**) wear profile and (**b**) contact pressure on the contact surface in 2000 loading cycles under fatigue loading $\sigma_{B}=450$ MPa.

Fatigue loading, residual stress, and contact stress are three important factors influencing the fretting fatigue crack initiation behavior of shot-peened specimens. The distributions of initial residual stress introduced by shot peening with different intensities are different. Usually, a higher shot peening intensity has a larger initial residual compressive stress and a thicker material layer with residual compressive stress. In addition to initial residual stress, shot peening also plays an important role in the distribution of contact stress. This is because the shot peening intensity determines the wear resistance of materials, and the wear resistance can affect the evolution of wear profile. Under the same loading conditions, the distribution of contact stress is determined by the wear profile. Taking 2000 fretting cycles as an example, Figure 9a shows the wear profile on the contact surface under sinusoidal fatigue loading $\sigma_{B}=450$ MPa with stress ratio R=−1 and constant normal pressure $P_{c}=4$ MPa. In this study, in order to analyze the influence of shot peening, two kinds of shot peeing intensities, namely 0.1 mmA and 0.2 mmA, are adopted in the calculation. In Figure 9, the origin of the abscissa represents the contact center, the positive direction of abscissa represents the specimen surface close to the fatigue loading, and the negative direction represents the specimen surface close to the fixed constraint. Due to lower wear resistance, the width and depth of the wear profile for the unpeened specimen are the largest and those for the 0.2 mmA shot-peened specimen are the smallest. The wear profile represents the fretting contact region. The larger contact region means that each node on the contact surface withstands less contact stresses. Contact stresses can be divided into contact pressure and contact shear stress. As shown in Figure 9b, it can be seen that in the same loading cycle, the contact pressure of the unpeened specimen is lowest, that of the 0.1 mmA shot-peened specimen is higher, and that of the 0.2 mmA shot-peened specimen is the highest. According to the wear profile, it can be judged that fretting is in a gross slip state. Thus, the contact shear stress can be approximately equal to contact pressure times the friction coefficient. Therefore, the specimen with higher shot peening intensity has larger contact shear stress in the same loading cycles. Moreover, it can be seen that the contact stresses of all cases are much lower than those of the initial state. Contact stresses are important factors causing stress concentration under fretting conditions. The decrease of contact stresses can reduce stress concentration, thereby reducing fretting damage. Therefore, the life prediction results without considering contact stress evolution will underestimate the crack initiation life of specimens.

**Figure 10:**
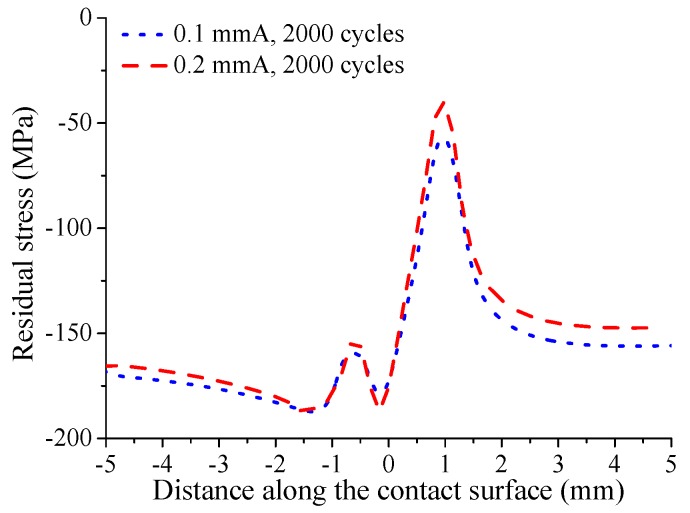
Distribution of average residual stress (by using the process volume method) in 2000 loading cycles under fatigue loading $\sigma_{B}=450$ MPa.

For shot-peened specimens, the relaxation of residual stress can weaken the effect of shot peening on the improvement of crack initiation life. In this study, when analyzing the crack initiation behavior, the average residual stress in a certain volume range is considered. As shown in Figure 10, by using the process volume approach, the distribution of average residual stress in volume range with 80 μm radius on the contact surface at 2000 cycles is obtained. It can be seen that the remaining residual compressive stress near the positive direction contact edge reaches the minimum value. The reason is that the Mises stress reaches its maximum near the positive direction contact edge due to the superposition of fatigue compressive stress, initial residual compressive stress, and contact stress. Moreover, although the initial average residual stress is larger for the specimen with high shot peening intensity, i.e., 0.2 mmA, the remaining residual stress is larger for the specimen with low shot peening intensity, i.e., 0.1 mmA. This is because the specimen with high shot peening intensity undergoes larger Mises stress due to larger initial residual compressive stress during a loading cycle. Furthermore, the in-plane tensile deformation caused by shot peeing can increase the tensile yield strength of the shot-peened layer, which will cause lower compressive yield strength [17]. The nano-indentation experiment results show that the 0.2 mmA shot-peened specimen has the larger tensile yield strength. Thus, it can be concluded that the specimen with high shot peening intensity has the lower compressive yield strength. With the higher Mises stress and the lower compressive yield strength, the residual compressive stress of the specimen with high shot peening intensity is more prone to relaxation.

**Figure 11:**
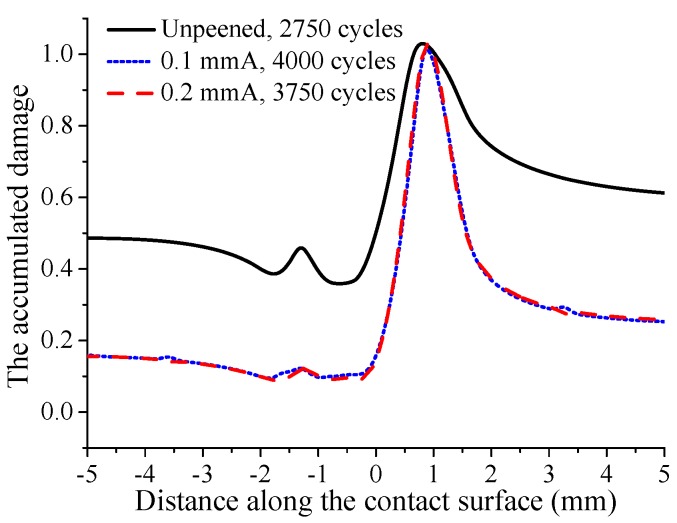
The accumulated damage on the contact surface after material failure under fatigue loading $\sigma_{B}=450$ MPa.

**Figure 12:**
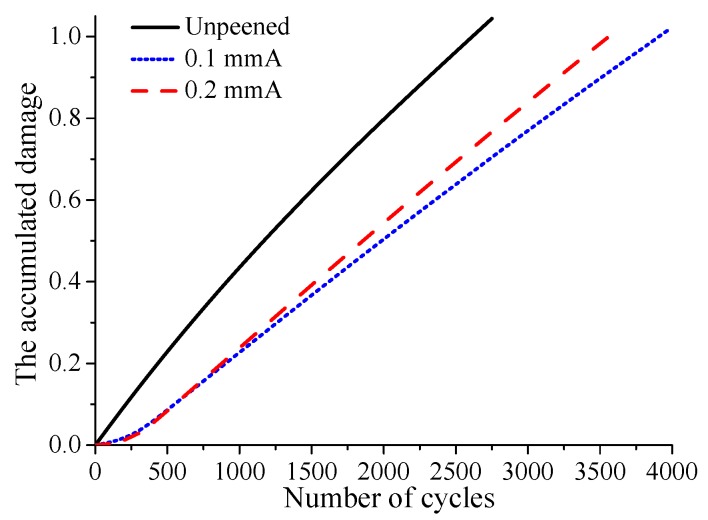
The evolution of accumulated damage at the crack initiation location with increasing cycle number under fatigue loading $\sigma_{B}=450$ MPa.

The evolutions of residual stress and contact stress are both considered in the prediction of crack initiation life. The calculation results show that under fatigue loading $\sigma_{B}=450$ MPa, the crack initiation life of the unpeened specimen is 2750, while that of 0.1 mmA shot-peening specimen is 4000, and shot peening improves the life of the specimen by 45%. The crack initiation life of the 0.2 mmA shot-peened specimen is 3750, and shot peening improves the life of the specimen by 36%. It can be seen that under the current fatigue loading, the specimen with lower shot peening intensity has a larger crack initiation life. A similar phenomenon can be found in M. Benedetti’s reverse bending fatigue experiments [30]. After crack initiation, the distribution of accumulated damage on the contact surface under larger fatigue loading $\sigma_{B}=450$ MPa is shown in Figure 11. As shown in the figure, the location where the cumulative damage reaches 1 is the crack initiation location. It can be found that the crack initiation locations of shot-peened and unpeened specimens are basically the same. This indicates that shot peening has little effect on the crack initiation location. In addition, although the crack initiation lives of the 0.1 mmA and 0.2 mmA shot-peened specimens are higher than those of unpeened specimen, the accumulated damage of shot-peened specimens on the contact surface except crack initiation location is much lower than that of unpeened specimen. Surface damage evolves gradually with increasing cycle number. At the crack initiation location, the relationship between the accumulated surface damage and cycle number is basically linear for all cases, as shown in Figure 12. Furthermore, it can be seen that the damage accumulation rate for unpeened specimens is obviously larger than that for shot-peened specimens, and the damage accumulation rate for the specimen with high shot peening intensity is slightly larger than that for the specimen with low shot peening intensity.

**Figure 13:**
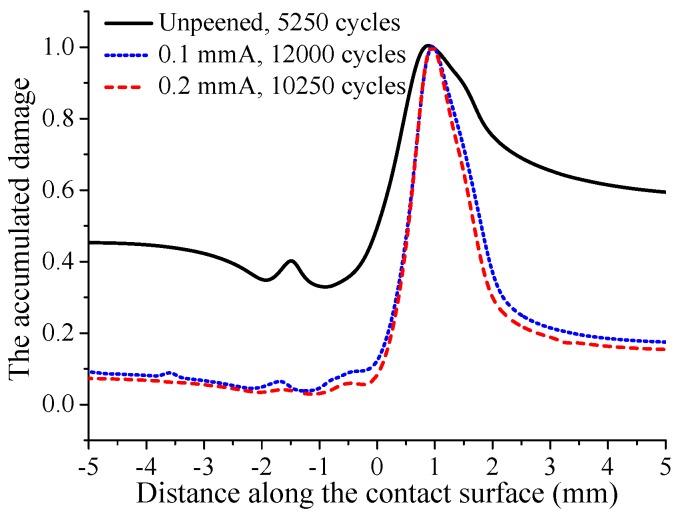
The accumulated damage on the contact surface after material failure under fatigue loading $\sigma_{B}=400$ MPa.

**Figure 14:**
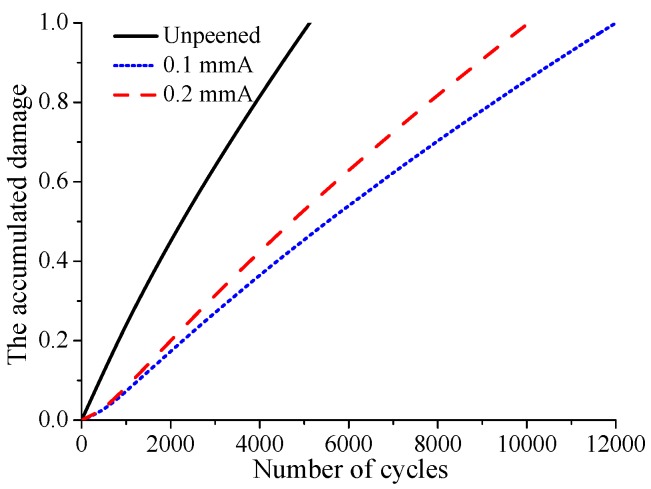
The evolution of accumulated damage at the crack initiation location with increasing cycle number under fatigue loading $\sigma_{B}=400$ MPa.

For the fatigue loading 400 MPa, a similar distribution of the accumulated damage on the contact surface to that for the larger fatigue loading 450 MPa is observed, as shown in Figure 13. But the difference is that the accumulated damage at other locations, except crack initiation location, for the 0.1 mmA shot-peened specimen is slightly larger that for 0.2 mmA. In addition, the results show that 0.1 mmA and 0.2 mmA shot peening increase the crack initiation life by 128% and 95%, respectively. Compared with the larger fatigue loading 450 MPa, it can be concluded that the effect of shot peening on the improvement of crack initiation life is more obvious for the smaller fatigue loading. Figure 14 shows the evolution of accumulated damage at the crack initiation location under 400 MPa. Like larger fatigue loading, there is also a linear relationship between accumulated damage and cycle number. However, unlike larger fatigue loading, the damage accumulation rates of the two shot-peened specimens show a greater difference.

**Figure 15:**
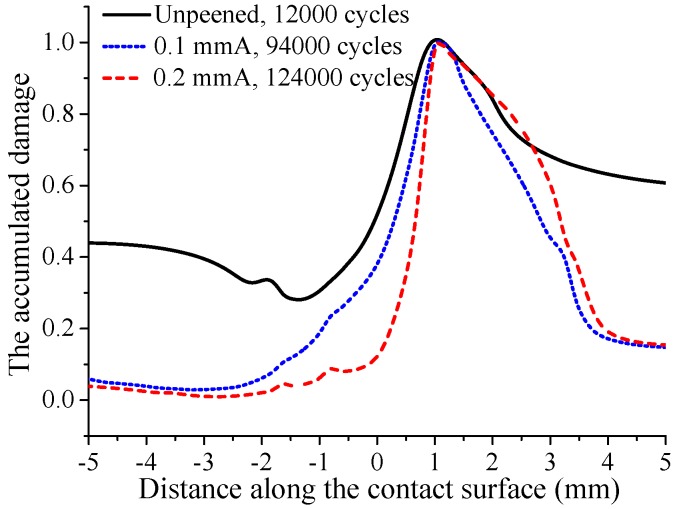
The accumulated damage on the contact surface after material failure under fatigue loading $\sigma_{B}=350$ MPa.

**Figure 16:**
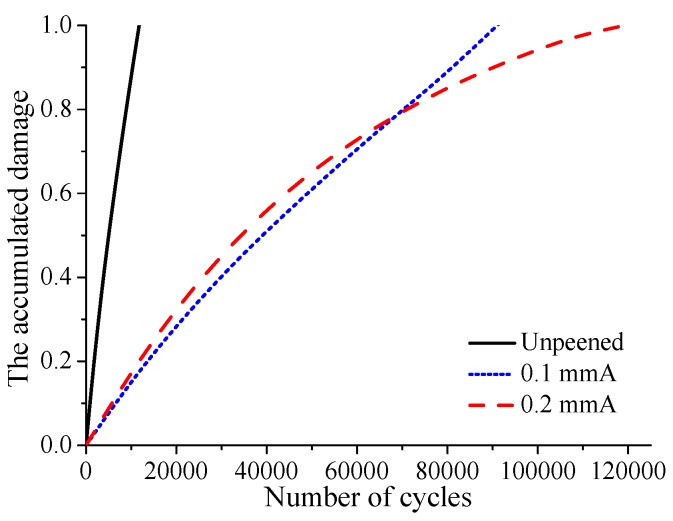
The evolution of accumulated damage at the crack initiation location with increasing cycle number under fatigue loading $\sigma_{B}=350$ MPa.

**Figure 17:**
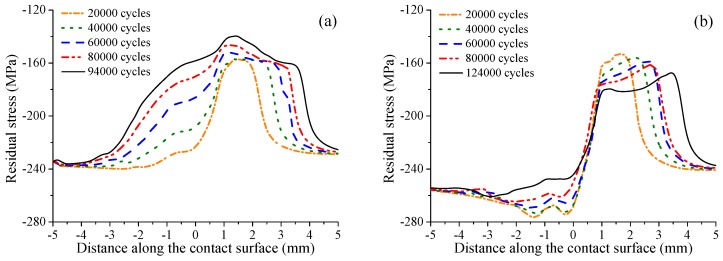
Distribution of average residual stress (by using the process volume method) on the contact surface for (**a**) 0.1 mmA and (**b**) 0.2 mmA shot-peened specimens under fatigue loading $\sigma_{B}=350$ MPa.

Figure 15 shows the distribution of accumulated damage on the contact surface after crack initiation under smaller fatigue loading i.e., $\sigma_{B}=350$ MPa. It can be seen that the crack initiation locations are similar to those under the larger fatigue loading, i.e., 400, 450 MPa. However, unlike the larger fatigue loading, the crack initiation life is longer for the specimen with high shot peening intensity. With low shot peening intensity, i.e., 0.1 mmA, the crack initiation life is increased by about 6.8 times. Meanwhile, with high shot peening intensity, i.e., 0.2 mmA, the crack initiation life is increased by up to 9.3 times. Figure 16 shows the evolution of surface damage at the crack initiation location. Like larger fatigue loading, the rate of damage accumulation for unpeened specimens is obviously larger than that for shot-peened specimens. But the difference is that the rate of damage accumulation for the specimen with high shot peening intensity is not always larger than that for the specimen with low shot peening intensity. After 60,000 fretting cycles, the damage accumulation rate for the 0.2 mmA shot-peened specimen is less than that for the 0.1 mmA shot-peened specimen. The remaining residual compressive stress is an important factor affecting the rate of damage accumulation. As shown in Figure 17, it can be seen that the magnitude of average residual compressive stress decreases with the increase of cycle number for 0.1 mmA shot-peened specimen. However, for the 0.2 mmA shot-peened specimen, the magnitude of average residual compressive stress near the crack initiation location increases with the increase of the number of loading cycles. After 60,000 fretting cycles, the magnitude of average residual compressive stress near the crack initiation location for the 0.2 mmA shot-peened specimen is larger than that for the 0.1 mmA shot-peened specimen. In addition, it can be seen that under smaller fatigue loading, the remaining residual compressive stress near the crack initiation location is greater than that under larger fatigue loading. This also explains why the effect of shot peening on life improvement is more obvious under smaller fatigue loading.

**Figure 18:**
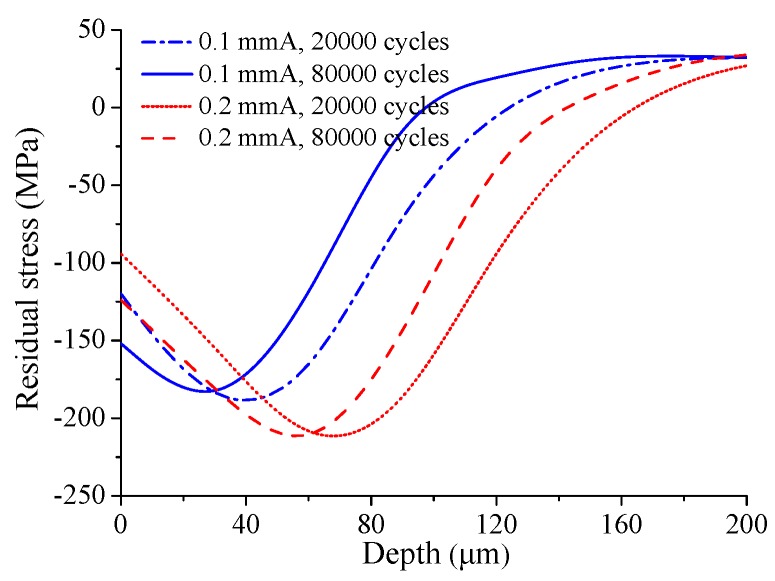
Residual stress versus the material layer depth with 20,000 and 80,000 cycles for different shot-peened cases.

Under fretting loading, the newly formed plastic deformation on the contact surface can cause relaxation of the initial residual compressive stress. However, due to the lower remaining residual compressive stress after relaxation, it is difficult to produce new plastic deformation at the same location in subsequent loading cycles. In addition to plastic deformation, the fretting wear behavior is also a factor affecting the distribution of residual stress. Fretting wear can cause material removal on the contact surface, and the volume of removed material increases with the increase of cycle number in the depth direction. The initial residual compressive stress introduced by shot peening increases first and then decreases in the depth direction, as shown in Figure 1. Thus, the subsurface with higher residual compressive stress becomes a newly formed contact surface. Taking 20,000 and 80,000 fretting cycles as examples, the residual stress distribution in the depth direction at the crack initiation location is shown in Figure 18. It can be seen from the figure that the distribution of residual stress at 20,000 cycles is basically parallel to that at 80,000 cycles. However, due to the fretting wear, the curve of 80,000 cycles shifts towards the contact surface relative to the curve of 20,000 cycles. In this study, the radius of the process volume method is 80 μm. By comparing the curves in the figure, it can be found that the overall distribution of residual compressive stress at 80,000 cycles is lower than that at 20,000 cycles in the range of 80 μm from the surface for the specimen with low shot peening intensity 0.1 mmA. However, the specimen with high shot peening intensity 0.2 mmA experiences an opposite phenomenon. This is the reason why the average residual compressive stress for the specimen with high shot peening intensity increases with an increasing number of loading cycles.

Finally, the predicted crack initiation lives versus the fatigue loadings are summarized in Table 4. It can be seen that the crack initiation lives of the shot-peened specimens are longer than those of the unpeened specimens under all fatigue loadings, and the smaller fatigue loading has the longer crack initiation life. Moreover, under smaller fatigue loading, i.e., 350 MPa, the high shot peening intensity improves crack initiation life more, while under larger fatigue loading the low shot peening intensity has a larger improvement on the crack initiation life. These findings indicate that improving shot peening intensity does not necessarily have a beneficial effect on crack initiation life, and then emphasize the importance of remaining residual stress after relaxation in the prediction of crack initiation life under fretting conditions. Furthermore, it is possible to minimize the relaxation of residual stress by choosing proper shot peening according to the loading conditions experienced by mechanical components, so as to maximize the effect of shot peening on the life improvement.

## 5. Conclusions

The application of the FE model in this study provides a detailed understanding of the effects of shot peening on the crack initiation life under fretting loading. The large stress gradient variation in the contact area and the evolution of contact stress and residual stress during fretting are taken into account in the FE model.

The current investigation finds that the remaining average residual stress after relaxation greatly determines the effect of shot peening on the improvement of crack initiation life. Furthermore, with increasing fatigue loading, the remaining average residual stress near the crack initiation location decreases, which leads to the weakening of the effect of shot peening on the improvement of crack initiation life.

The effect of shot peening intensity on crack initiation life is more complex. Under smaller fatigue loading 350 MPa, the higher intensity shot peening is more effective in improving the crack initiation life. In this study, the 0.1 mmA shot peening only increases the crack initiation life by 6.8 times, while the 0.2 mmA shot peening increases the crack initiation life by up to 9.3 times.

Under relatively larger fatigue loading, the remaining average residual stress near the crack initiation location for the specimen with low shot peening intensity is larger than that for the specimen with high shot peening intensity. Therefore, the lower intensity shot peening is more effective in improving the crack initiation life. In the studied cases with the largest fatigue loading 450 MPa, the 0.1 mmA shot peening increases the crack initiation life by 45%, while the 0.2 mmA shot peening only increases the crack initiation life by 36%. This indicates that under larger fatigue loading, increasing shot peening intensity does not necessarily have a beneficial effect on fatigue life.

## Figures and Tables

**Table 1:** Material properties of aluminum 7075-T6.

Young’s Modulus *E*	Poisson’s Ratio ν	Yield Strength $\sigma_{y}$	Tensile Strength $\sigma_{b}$
72,000 MPa	0.345	545 MPa	654 MPa

**Table 2:** Values of coefficients in Equation (1).

Cases	$a_{1}$	$b_{1}$	$c_{1}$	$a_{2}$	$b_{2}$	$c_{2}$	$a_{3}$	$b_{3}$	$c_{3}$	$a_{4}$	$b_{4}$	$c_{4}$
0.1 mmA	1265	0.1368	0.0245	1121	0.0226	2.127	409.8	0.0289	4.519	0	0	0
0.2 mmA	1009	0.0104	−0.0314	565.9	0.0190	1.746	280.7	0.0362	2.089	172	0.0401	4.574

**Table 3:** Material parameters of the Smith–Watson–Topper (SWT) model for aluminum 7075-T6 [25].

$\sigma_{f}^{\prime }$ (MPa)	$\varepsilon_{f}^{\prime }$	*b*	*c*
1466	0.262	−0.143	−0.619

**Table 4:** Predicted crack initiation life for different fatigue loadings.

Cases	Unpeened	0.1 mmA	0.2 mmA
$\sigma_{B}=350$ MPa	12,000	94,000	124,000
$\sigma_{B}=400$ MPa	5250	12,000	10,250
$\sigma_{B}=450$ MPa	2750	4000	3750
